# Supplementary figures and images for: PIP-seq identifies novel heterogeneous lung innate lymphocyte population activation after combustion product exposure
Source: Sci Rep. 2024 Aug 30;14:20167. doi: 10.1038/s41598-024-70880-y (PMC11364781; doi:10.1038/s41598-024-70880-y)

Figure S1

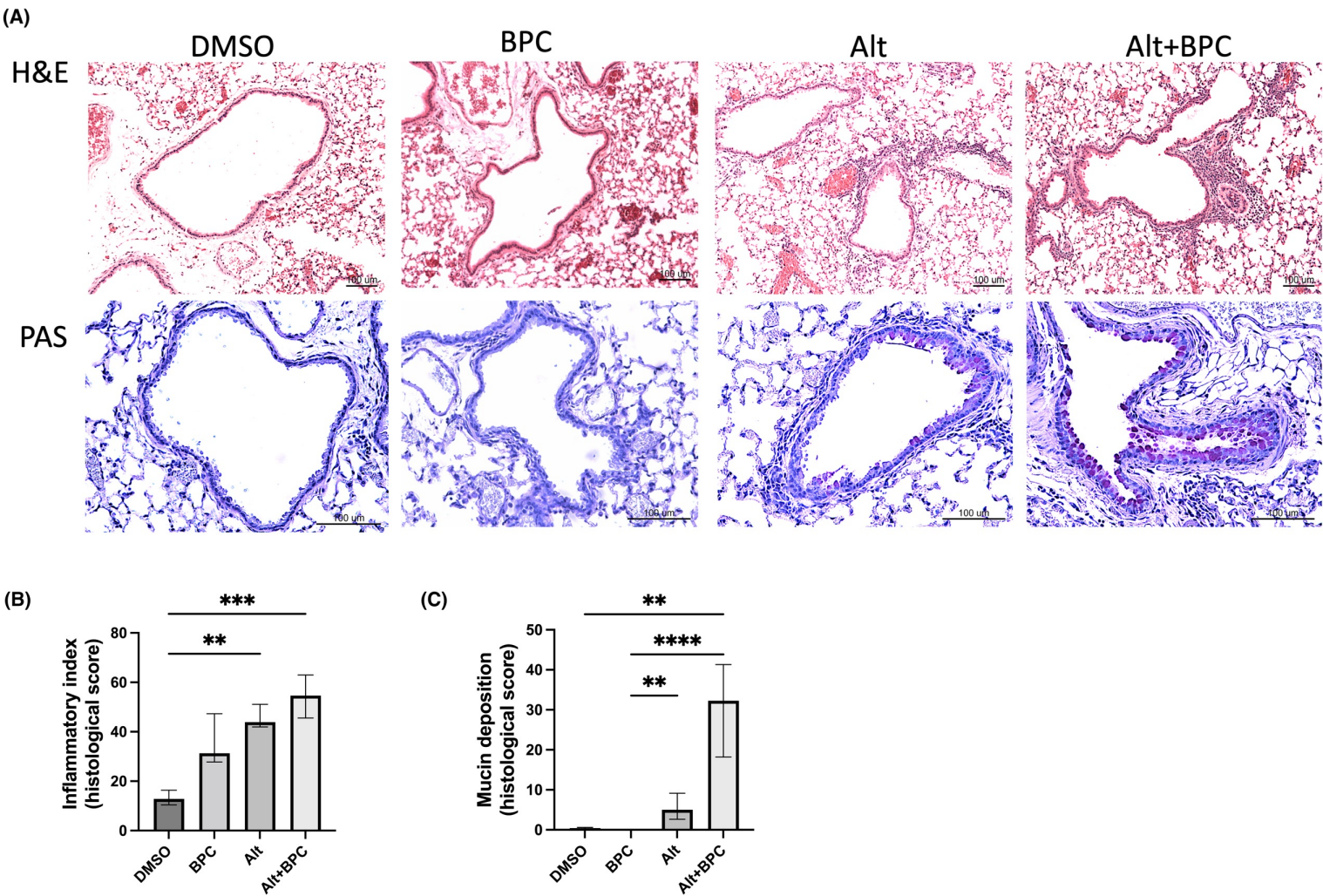

Figure S2

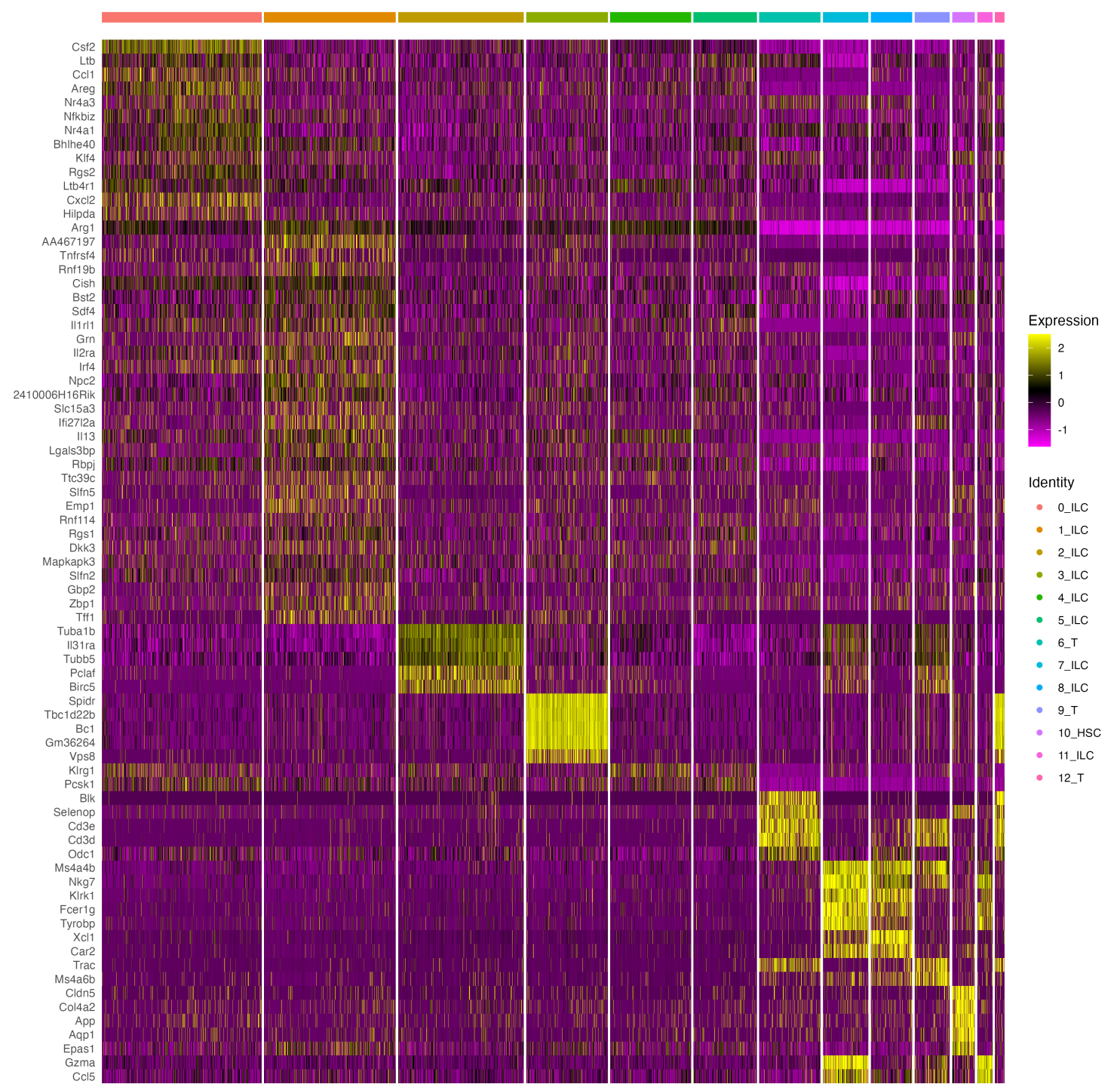

Figure S3

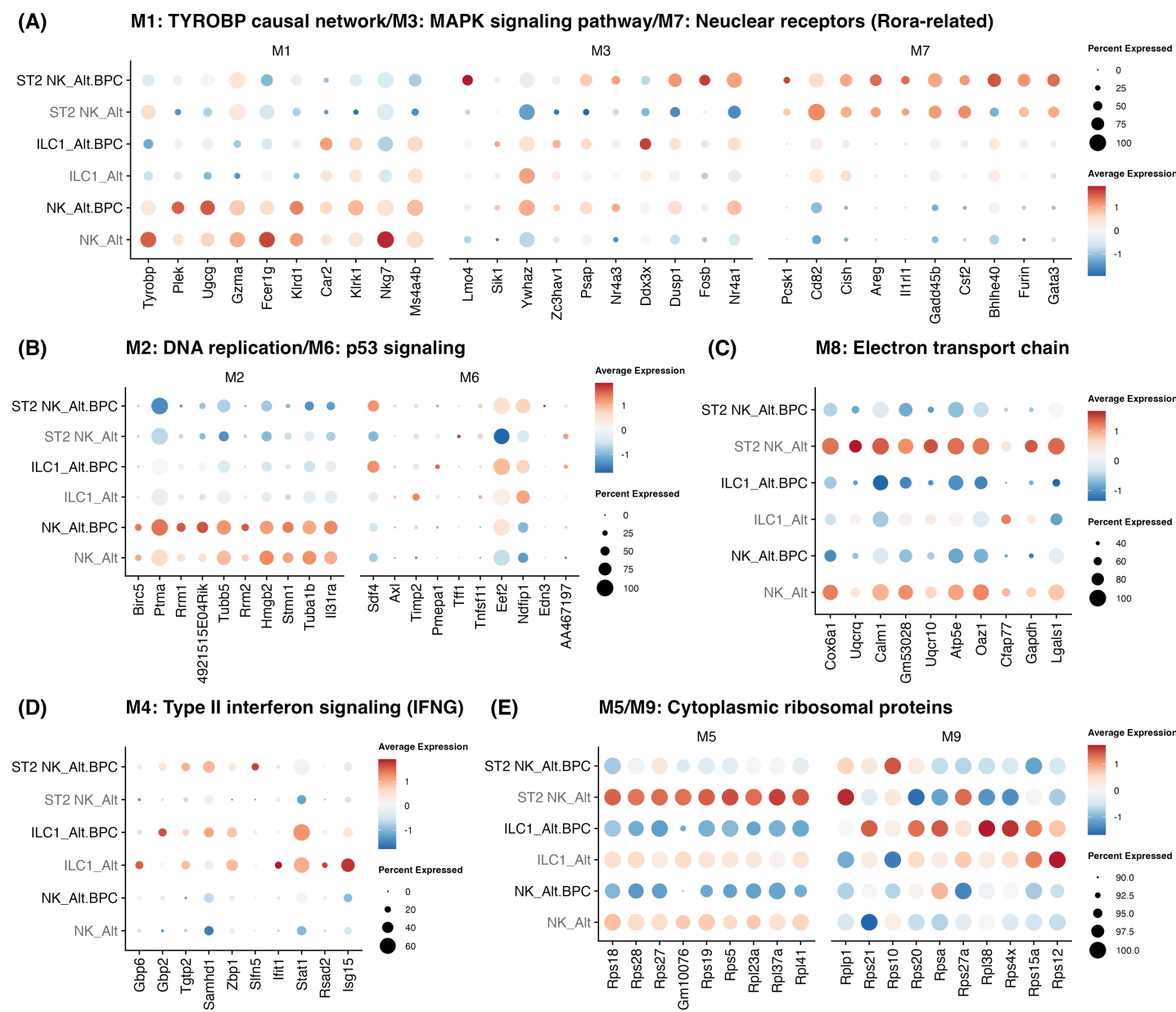

Figure S4

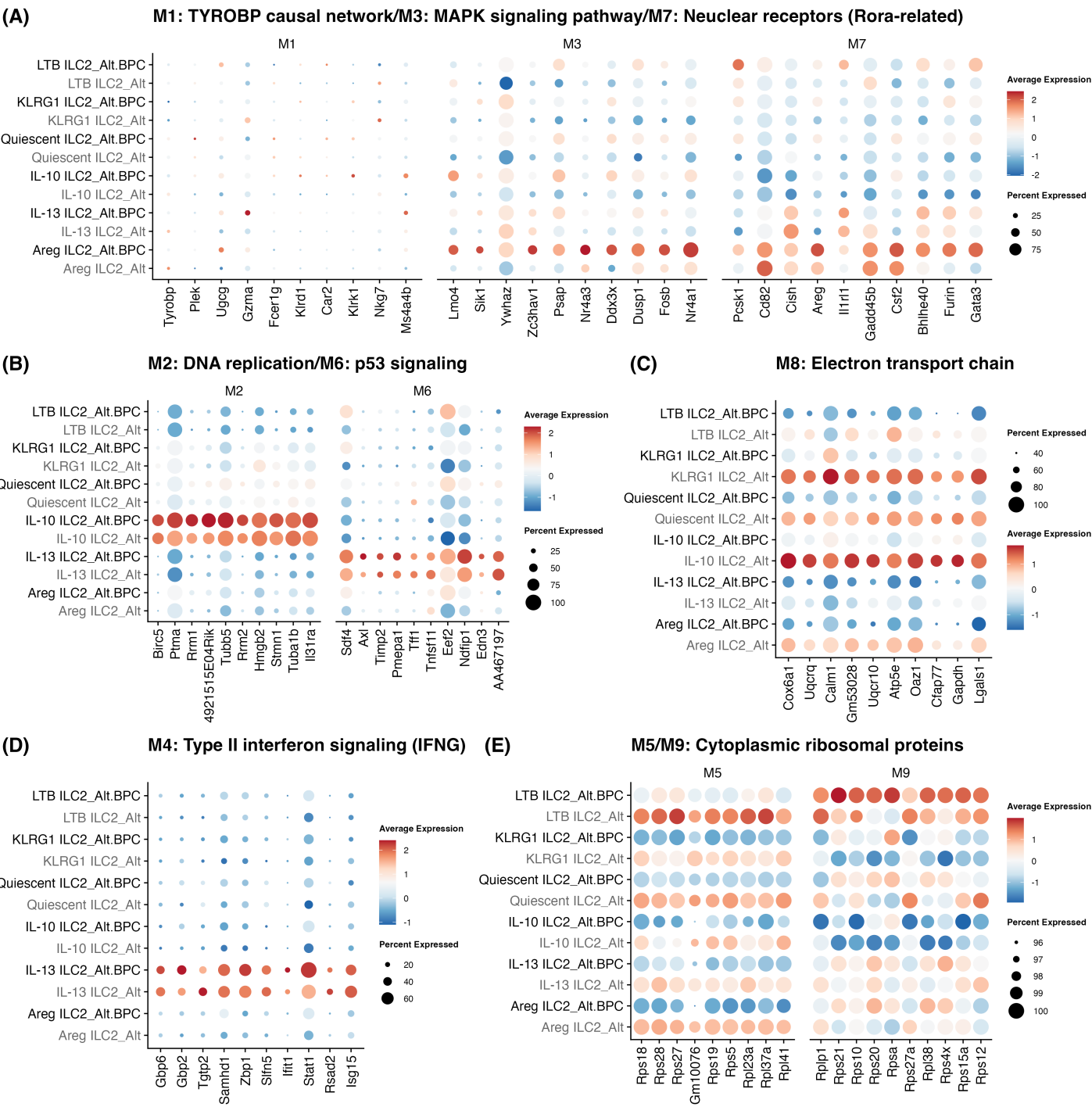

Figure S5

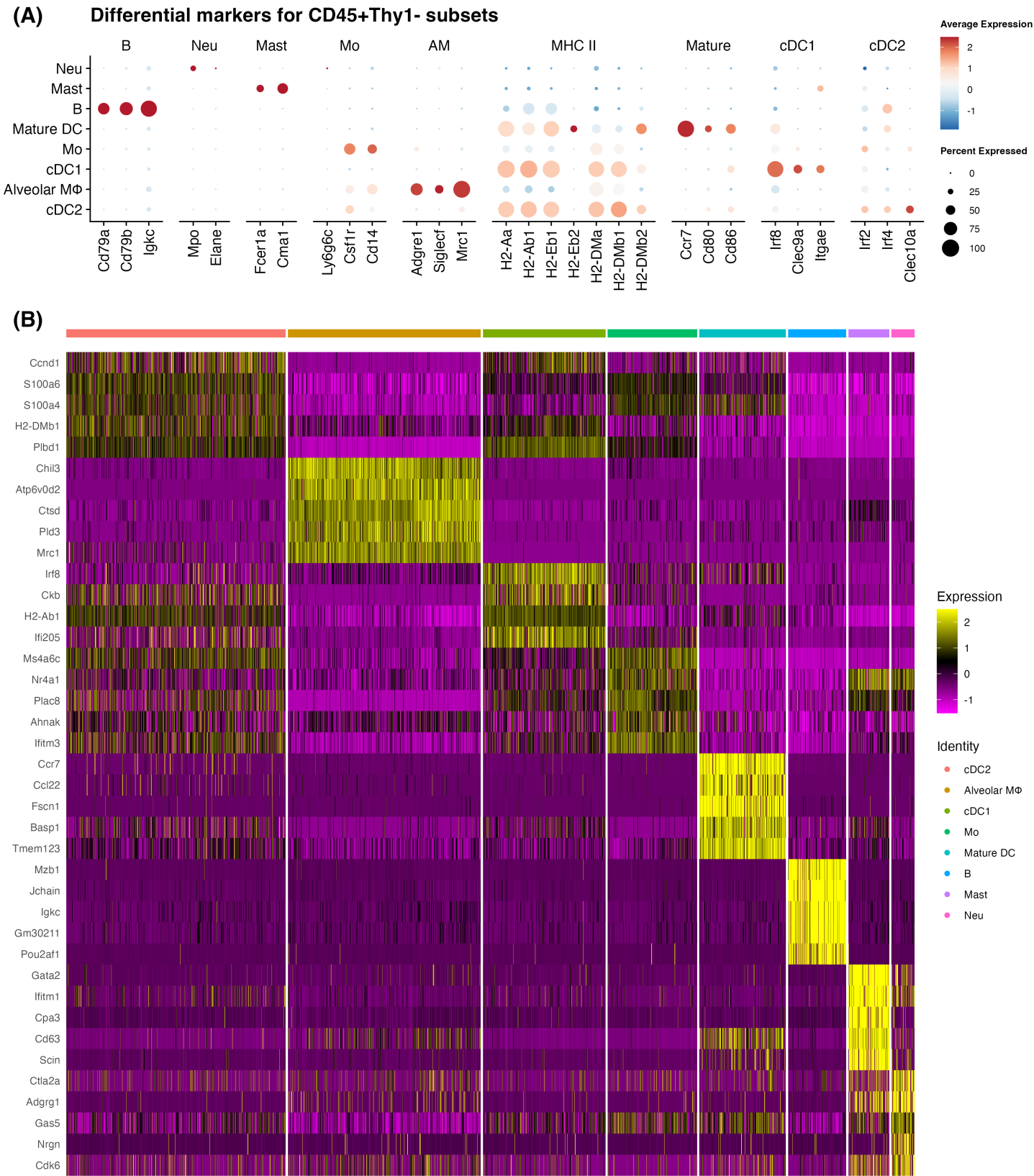

Figure S6

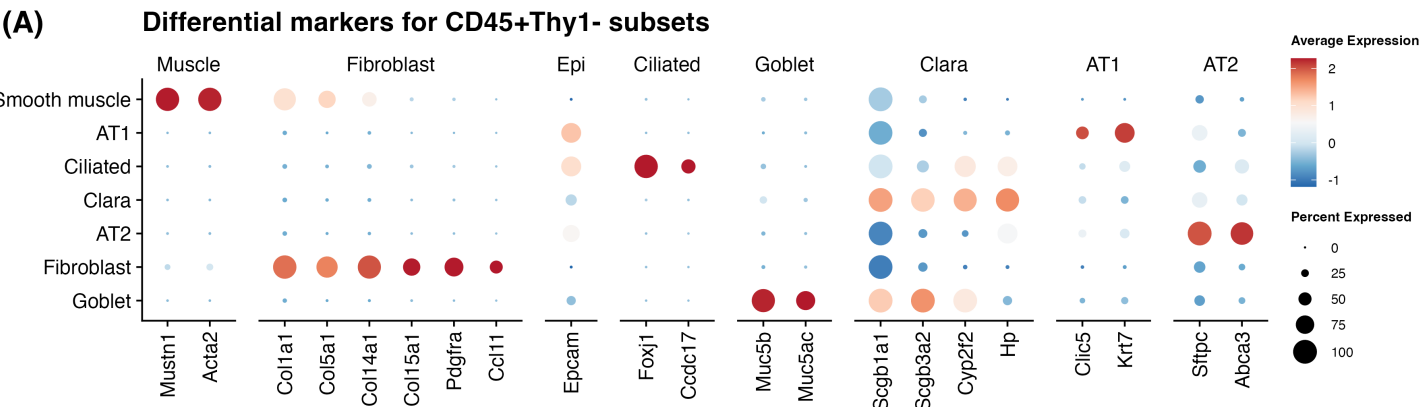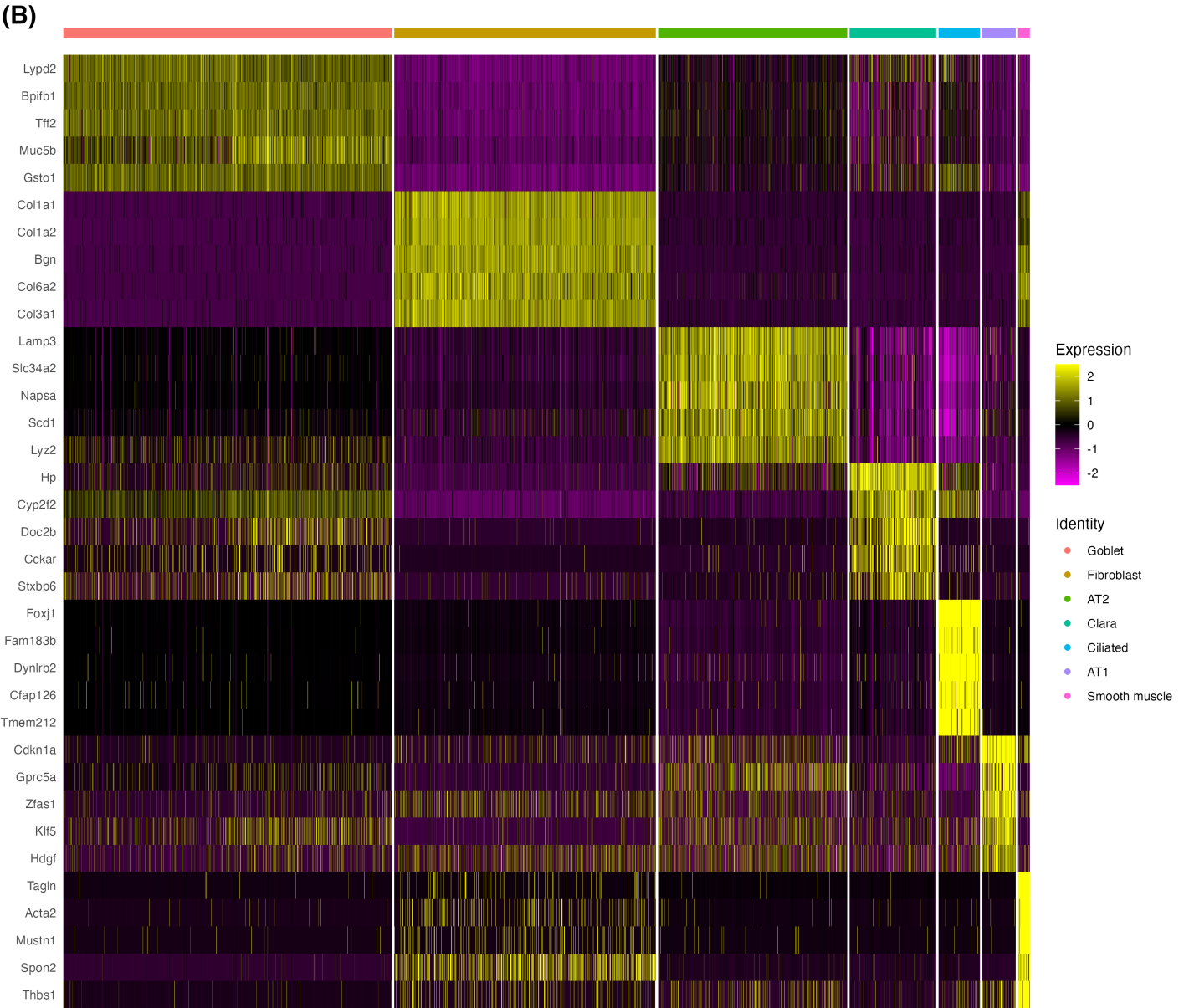

Supplement: Supplementary file 1 — Supplementary Figures. [file 41598_2024_70880_MOESM1_ESM.pdf]
