# Supplementary material for: PIP-seq identifies novel heterogeneous lung innate lymphocyte population activation after combustion product exposure
Source: Sci Rep. 2024 Aug 30;14:20167. doi: 10.1038/s41598-024-70880-y (PMC11364781; doi:10.1038/s41598-024-70880-y)
Supplement: Supplementary file 2 — Supplementary Legends. [file 41598_2024_70880_MOESM2_ESM.docx]

**Supplementary materials and methods**

**Lung histology**

The lungs without BALF collection were fixed with formaldehyde and stained (hematoxylin and eosin, H&E and periodic acid-Schiff, PAS), as previously described (ref PMID 35908044). H&E photos were captured at 10X magnification, and PAS photos were captured at 20X magnification. Five areas with 100 µm square size around the bronchiole in selected field were analyzed by ImageJ software (NIH) with Threshold_Color plugin. The quantitation of histological changes and mucin deposition was determined by HSB assay (hue: 182–242, saturation: 80–255, and brightness: 0–255 for H&E; and hue: 185–200, saturation: 100–255, and brightness: 0–255 for PAS). The mean intensity of each area was recorded as the histological score.

**Supplementary figure legends**

Figure S1. The formaldehyde-fixed, paraffin-embedded lung samples were sliced and stained by (A) hematoxylin and eosin (H&E) staining and periodic acid-Schiff (PAS) staining. The magnification folds are 10X for H&E and 20X for PAS. The quantitative data of each slide were analyzed by ImageJ and presented as histological score, including (B) inflammatory index and (C) mucin deposition. There were five areas in three fields applied for quantification. Scale bar = 100 µm. Data are presented as median with interquartile (**p < 0.01, ***p < 0.001, ****p < 0.0001).

Figure S2. Marker genes for each cluster in CD45^+^Thy1^+^ cell subsets are illustrated as expression level in heat map form.

Figure S3. Top 10 hub gene expression from each module in ILC1 and NK are shown in dot plots.

Figure S4. Top 10 hub gene expression from each module in ILC2 are shown in dot plots.

Figure S5. The feature genes (A) for B cell, neutrophil (Neu), Mast cell, monocyte (Mo), alveolar macrophage (AM), MHC class II, type 1 conventional dendritic cell (cDC1), cDC2, mature DC (mature) were used to identify the populations of CD45^+^Thy1^-^ leukocytes. Marker genes for each cluster in CD45^+^Thy1^-^ cell subsets (B) are illustrated as expression level in heat map form.

Figure S6. The feature genes (A) for muscle, fibroblast, epithelial (Epi), lung ciliated cell, goblet cell, lung Clara cell, type 1 alveolar cell (AT1), and type 2 alveolar cell (AT2) were used to identify the populations of CD45^-^ cells. Marker genes for each cluster in CD45^-^ cell subsets (B) are illustrated as expression level in heat map form.
